# miR-124 and miR-137 inhibit proliferation of glioblastoma multiforme cells and induce differentiation of brain tumor stem cells

**DOI:** 10.1186/1741-7015-6-14

**Published:** 2008-06-24

**Authors:** Joachim Silber, Daniel A Lim, Claudia Petritsch, Anders I Persson, Alika K Maunakea, Mamie Yu, Scott R Vandenberg, David G Ginzinger, C David James, Joseph F Costello, Gabriele Bergers, William A Weiss, Arturo Alvarez-Buylla, J Graeme Hodgson

**Affiliations:** 1 Department of Neurological Surgery, University of California San Francisco, San Francisco, CA, USA; 2 Department of Neurology, University of California San Francisco, San Francisco, CA, USA; 3 Department of Pediatrics, University of California San Francisco, San Francisco, CA, USA; 4 Department of Pathology, University of California San Francisco, San Francisco, CA, USA; 5 Applied Biosystems, Foster City, CA, USA

## Abstract

**Background:**

Glioblastoma multiforme (GBM) is an invariably fatal central nervous system tumor despite treatment with surgery, radiation, and chemotherapy. Further insights into the molecular and cellular mechanisms that drive GBM formation are required to improve patient outcome. MicroRNAs are emerging as important regulators of cellular differentiation and proliferation, and have been implicated in the etiology of a variety of cancers, yet the role of microRNAs in GBM remains poorly understood. In this study, we investigated the role of microRNAs in regulating the differentiation and proliferation of neural stem cells and glioblastoma-multiforme tumor cells.

**Methods:**

We used quantitative RT-PCR to assess microRNA expression in high-grade astrocytomas and adult mouse neural stem cells. To assess the function of candidate microRNAs in high-grade astrocytomas, we transfected miR mimics to cultured-mouse neural stem cells, -mouse oligodendroglioma-derived stem cells, -human glioblastoma multiforme-derived stem cells and -glioblastoma multiforme cell lines. Cellular differentiation was assessed by immunostaining, and cellular proliferation was determined using fluorescence-activated cell sorting.

**Results:**

Our studies revealed that expression levels of microRNA-124 and microRNA-137 were significantly decreased in anaplastic astrocytomas (World Health Organization grade III) and glioblastoma multiforme (World Health Organization grade IV) relative to non-neoplastic brain tissue (P < 0.01), and were increased 8- to 20-fold during differentiation of cultured mouse neural stem cells following growth factor withdrawal. Expression of microRNA-137 was increased 3- to 12-fold in glioblastoma multiforme cell lines U87 and U251 following inhibition of DNA methylation with 5-aza-2'-deoxycytidine (5-aza-dC). Transfection of microRNA-124 or microRNA-137 induced morphological changes and marker expressions consistent with neuronal differentiation in mouse neural stem cells, mouse oligodendroglioma-derived stem cells derived from S100β-v-*erbB *tumors and cluster of differentiation 133+ human glioblastoma multiforme-derived stem cells (SF6969). Transfection of microRNA-124 or microRNA-137 also induced G1 cell cycle arrest in U251 and SF6969 glioblastoma multiforme cells, which was associated with decreased expression of cyclin-dependent kinase 6 and phosphorylated retinoblastoma (pSer 807/811) proteins.

**Conclusion:**

microRNA-124 and microRNA-137 induce differentiation of adult mouse neural stem cells, mouse oligodendroglioma-derived stem cells and human glioblastoma multiforme-derived stem cells and induce glioblastoma multiforme cell cycle arrest. These results suggest that targeted delivery of microRNA-124 and/or microRNA-137 to glioblastoma multiforme tumor cells may be therapeutically efficacious for the treatment of this disease.

## Background

MicroRNAs (miRNAs) are a class of small non-coding RNAs that regulate diverse cellular processes through RNA interference-based mechanisms. miRNAs are transcribed as primary RNA transcripts (pri-miRNAs), processed in the nucleus to smaller precursor hairpin structures (pre-miRNAs), and then exported to the cytoplasm where they are processed further by the Dicer nuclease to become mature, functional miRNAs approximately 21 nucleotides in length. Mature miRNAs, the endogenous equivalent of short interfering RNAs (siRNAs), are then incorporated into the RNA-induced silencing complex, which facilitates their interaction with, and inhibition of, target messenger RNAs (mRNAs) by translational repression or message cleavage (as reviewed in [1]).

Since the initial discovery of miRNAs as developmental mutants in *Caenorhabditis elegans*, their role as important regulators of stem cell division and development in evolutionarily divergent organisms has become increasingly apparent. For example, functional ablation of the RNaseIII enzyme Dicer, resulting in the ablation of miRNA biogenesis, disrupts the division of insect germline stem cells [2] and mouse embryonic stem cells [3], and impairs early embryonic development in zebrafish [4] and mice [5]. It is also becoming increasingly evident that miRNAs play important roles in cancer etiology. For example, expression of the mir-17–92 miRNA cluster augments tumor angiogenesis [6] and accelerates c-Myc-induced B-cell lymphoma development in mice [7], and the let-7 miRNA transcriptionally regulates the *ras *oncogene [8] and inhibits growth of lung adenocarcinoma cells [9]. Further, impairment of miRNA processing enhances cellular transformation and tumorigenesis [10], consistent with observations that global down-regulation of miRNAs occurs in multiple tumor types compared with normal tissues [11].

Most recently, specific miRNAs have been implicated in the differentiation of cultures derived from mouse embryonic stem (ES) cells and mouse tumors. For example, expression of miR-124 and miR-9 increases during differentiation of mouse ES cell-derived neural progenitors, and experimental manipulation of miR-124 and miR-9 expression affects neural lineage differentiation in the ES cell-derived cultures [12]. Up-regulation of miR-124 also induces neuronal differentiation of mouse neuroblastoma cell lines CAD and Neuro2a and the mouse embryonal tumor cell line P19 [13]. These results suggest that miRNAs may be valuable therapeutic agents if they similarly promote differentiation of human tumor cells and tumor stem cells (TSCs).

The discovery of a rare, highly tumorigenic, self-renewing sub-population of glioblastoma multiforme (GBM) cells that express the cell surface marker cluster of differentiation (CD) 133 (see [14,15]), the so-called GBM stem cell population, suggests that therapeutic approaches that effectively inhibit or kill CD133+ GBM stem cells may lead to marked improvements in patient outcome. To this end, it has recently been demonstrated that induction of differentiation of CD133+ GBM cells by bone morphogenetic protein 4 can effectively inhibit intracerebral GBM tumor growth in mice [16]. In light of the growing body of evidence supporting a role for miRNAs in promoting stem cell differentiation, we investigated the role of miRNAs in differentiation and proliferation of human GBM stem cells, mouse oligodendroglioma tumor stem cells (mOSCs) and normal adult mouse neural stem cells (mNSCs), putative progenitors of adult gliomas [17]. Our results show that miR-124 and miR-137 can induce neuronal differentiation of OSCs and GBM stem cells and inhibit proliferation of GBM cell lines. These results suggest that miR-124 and miR-137 may be useful therapeutic agents for the treatment of GBMs.

## Methods

### Primary human tissues

Fresh frozen primary human tissues were acquired from the Brain Tumor Research Center tissue core at the University of California San Francisco (UCSF) in accordance with the Committee on Human Research approved procedures. All samples were thoroughly reviewed by a neuropathologist (S Vandenberg), and anaplastic astrocytomas (AAs) and GBM tumors were confirmed to contain at least 90% tumor. Non-neoplastic brain tissues were derived from the temporal lobes of epileptic patient surgeries and comprised primarily cortex with mild to moderate reactive astrocytosis and neurons. For further details about the samples see Additional file 1.

### Quantitative reverse transcriptase polymerase chain reaction

Total RNA was extracted using the miR-Vana RNA isolation system (Ambion, Austin TX). Expression of 192 human miRNAs was quantitated in human tissues using TaqMan^® ^miRNA assays human panel-early access kit (Applied Biosystems, Foster City CA). Expression of the six high-grade astrocytomas (HGA)-miRNAs during NSC differentiation was quantitated using individual TaqMan^® ^MicroRNA Assays. The comparative Ct (ΔΔCt) method was used to determine the expression fold change.

### Statistical analyses

Statistical analysis of miRNA expression in primary tissues was performed on log2 transformed fold change data using freely available R language. The limma package in Bioconductor was used to compare the three types of primary tissues (glioses, AAs and GBMs). Moderated *t*-statistics were obtained as described elsewhere [18] and P-values were adjusted for multiple comparisons by controlling the false discovery rate. Changes were considered significant if the false discovery rate was less than 0.05.

### Demethylation and deacetylation experiments

U87 and U251 glioma cell lines were seeded at 1 × 10^5 ^cells per well of a six-well plate, incubated for 24 hours in Dulbecco's Modified Eagle's Medium (DMEM) high glucose 10% serum, and then supplemented with fresh media containing 5-aza-dC (1 or 5 μM; Sigma-Aldrich) for 72 hours or trichostatin A (TSA) (100 ng/ml; Sigma-Aldrich) for 12 hours. For the combination study, 1 or 5 μM 5-aza-dC was present for 72 hours and TSA was added for the last 12 hours. The media containing drugs were changed every 24 hours.

### miRNA oligonucleotides

miRIDIAN miRNA mimic negative control (cel-miR-67) and miRIDIAN miRNA mimics (mmu-miR-124, mmu-miR-137) were purchased from Dharmacon (Lafayette, CO) and validated using the pMIR-REPORT miRNA Expression Reporter Vector System (Ambion, Austin, TX). For results, see Additional file 2.

### CDK6-3'UTR miR-137 reporter assays

Cyclin-dependent kinase 6 (CDK6)-3'UTR reporter assays were performed in U251 cells. pMIR-REPORT vectors harboring CDK6-3'UTR sequences with wild type (WT) miR-137 binding sites or mutated (MUT) miR-137 binding sites were generated by cloning the following oligonucleotides into the *HindIII *and *SpeI *restriction sites of pMIR-REPORT: CDK6-UTR-WT fw 5'-AGCTTGATCACAGAAATATTGCTAGCTGATACATATTATTGCATTTCATAAAACTA CDK6-UTR-WT rv 5'-CTAGTAGTTTTATGAAATGCAATAATATGTATCAGCTAGCAATATTTCTGTGATCA CDK6-UTR-MUT fw 5'-AGCTTGATCACAGAAATTAACGAAGCTGATACATATTATTGCATTTCATAAAACTA CDK6-UTR-MUT rv 5'-CTAGTAGTTTTATGAAATGCAATAATATGTATCAGCTTCGTTAATTTCTGTGATCA Cells were transfected with (1) miR-137 or cel-miR-67-negative-control mimics (50 nM), (2) pMIR-REPORT vectors containing WT or MUT miR-137 binding sites (400 ng) and (3) pRL-SV40 (Promega) expressing *Renilla luciferase *(400 ng) for normalization. Cells were grown in high-glucose DMEM supplemented with 10% fetal bovine serum, and luciferase measurements were performed 48 hours post-transfection using the Dual-Luciferase Reporter Assay System (Promega).

### Establishment and transfection of mouse subventricular zone-NSCs

Adult mouse subventricular zone (SVZ)-NSC cultures were derived and grown as described previously [19] with a few modifications. SVZ microdissections from 2-month-old CD-1 mice (Charles River Laboratories) were dissociated to a single cell suspension with 0.25% trypsin, 0.5 mM ethylene diamine tetraacetic acid (EDTA) and gentle trituration. Cells were cleared on a 22% Percoll (Sigma) step-gradient (2) and grown in proliferation medium (DMEM/F12/N2), 5% fetal calf serum (FCS), 20 ng/ml epidermal growth factor (EGF), 20 ng/ml basic fibroblast growth factor (bFGF) and 35 μg/ml bovine pituitary extract (all media and supplements from Invitrogen, Inc.). Non-attached cells were collected after 1 day and replated into a 35-mm tissue culture dish (Corning). After 7 to 10 days, the plate was hyperconfluent with SVZ-NSCs and these were routinely passaged 1:2 with 0.25% trypsin and 0.5 mM EDTA. Cells were passaged at least six times before use in experiments. Media was half-changed every 2 days and completely changed every 4 days. Differentiation of SVZ-NSCs for the miRNA expression time course was induced by removing EGF, FGF and FCS from the media [19].

For transfection of miR-124/137 into SVZ-NSCs, 50,000 cells were plated into eight-well culture slides (BD Falcon Biosciences) pretreated with 0.1 mg/ml poly-D-lysine (Sigma) and 10 μg/ml laminin (Invitrogen) 24 hours prior to transfection. A total of 100 nM miRIDIAN miRNA mimics (50 nM each for miR-124 and miR-137 co-transfections) were complexed with LipofectAMINE 2000 (Invitrogen) and added directly to cells growing in proliferating medium. Transfection and proliferating medium was removed 12 to 24 hours post-transfection and cells were induced to differentiate as described above.

### Growth and transfection of S100βv-*erbB *mouse tumor stem cells

Adult tumor stem cells were derived from a low-grade oligodendroglioma of a 120-day-old FVB/N transgenic mouse expressing the v-*erbB *transgene under control of the S100β promoter [20]. Tumor tissue was microdissected from the surrounding normal brain and dissociated to a single cell suspension with papain, gentle trituration and filtration through a 40-μM mesh screen (Falcon). Neurospheres were grown from single cells in Neurobasal medium (Invitrogen) supplemented with 20 ng/ml EGF (Sigma), 20 ng/ml bFGF (Peprotech) and B27 (Invitrogen) on low-adherent tissue-culture dishes (Corning). After four passages, neurospheres were dissociated and replated into 10-cm culture dishes (Corning) in proliferation medium (see above). These TSCs are self-renewing and multi-potent and express markers for astrocytes (glial fibrillary acidic protein, GFAP), neuronal progenitors (Tuj1) and oligodendrocytic progenitors (NG2) under differentiating conditions.

For miRNA transfections, 25,000 cells were plated into eight-well pre-coated culture dishes (Nunc), 24 hours prior to transfection. Transfections and differentiation procedures were performed as described for SVZ-NSC cultures.

### Growth, CD133 sorting and transfection of early passage human GBM cells (SF6969)

Human GBM tissue was acquired by surgical removal after informed consent at UCSF and washed with Hank's buffered saline solution without magnesium and calcium. Tumors were then enzymatically dissociated with papain (Worthington) for 30 minutes at 37°C. After centrifugation and one wash with phosphate buffered saline, pH 7.4, cells were transferred to NBE media consisting of neurobasal media without retinoic acid (Invitrogen), N2 and B27 supplements (0.5× each; Invitrogen), 20 ng/ml human recombinant bFGF (Peprotech) and 20 ng/ml human recombinant EGF (Sigma-Aldrich). Cells were plated in ultra-low adherent plates (Corning). Medium was changed every 3 to 5 days.

Cells cultured in suspension were dissociated using accutase (Innovative Cell Technologies) for 30 minutes at 37°C. After one wash in RinseMACS buffer (Miltenyi Biotech), cells were incubated with magnetic beads conjugated with an antibody against the CD133/1 epitope. The cells were incubated with beads for 30 minutes at 4°C. Thereafter, cells were washed with 20× RinseMACS buffer, centrifuged and added on to large cell columns connected to a pre-separation filter. Fluorescence-activated cell sorter analyses confirmed a pure CD133- fraction and a highly enriched CD133+ fraction.

For transfections, both CD133+ and CD133- cells were plated (20,000 cells per well) in 24-well plates coated with polyornithine and laminin. Cells were transfected with miR-124 and/or miR-137 (100 nM) or a negative control oligonucleotide for 4 hours using lipofectamine. Cells were then washed and cultured for 10 days in NBE media without growth factors.

### Immunocytochemistry

Stem cell cultures were fixed, washed and preblocked prior to incubation with primary antibodies (Tuj1, 1:500, Covance Inc.; GFAP, rabbit polyclonal, 1:500, Dako Inc.; microtubule-associated protein 2 (MAP2) ab, 1:500, Sigma). Cells were then stained with Alexa488- or Alexa594-conjugated secondary antibodies and the nuclei counterstained with Hoechst 33258 (Molecular Probes) or DAPI (Sigma).

### Cell cycle analysis

Cell cycle analyses were conducted using the fluorescein isothiocyanate BrdU Flow Kit following manufacturer's recommendations (BD Pharmingen, San Diego, CA).

### Immunoblotting

Immunoblotting was performed using standard protocols with antibodies CDK6 (1:1000; Cell Signaling, Temecula, CA), Phospho-Rb (1:1000; Cell Signaling Technology, Temecula, CA), and β-actin (1:5000; Sigma, St Louis, MO).

For more detailed information regarding experimental methods, see Additional file 3.

## Results

### miR-124 and miR-137 are down-regulated in high-grade gliomas and up-regulated during adult NSC differentiation

To identify deregulated miRNAs that have not been previously implicated in GBM cells [21,22], we used quantitative reverse transcriptase polymerase chain reaction (RT-PCR; Taqman) to measure expression of 192 mature miRNA sequences in human non-neoplastic brain tissues (glioses), AAs (World Health Organization (WHO) grade III) and GBMs (WHO grade IV). The comparative Ct (ΔΔCt) method was used to determine the expression fold change of each miRNA in tumor samples relative to glioses (see Additional file 4). Briefly, the ΔCt of each miRNA was determined relative to let-7a and miR-16, endogenous control miRNAs that were robustly and invariantly expressed across all samples (see Additional file 5) and the average ΔCt of the four glioses was used as the calibrator for the tumor samples. Consistent with previous observations in GBMs, we observed recurrent up-regulation of miR-10b [22] and miR-21 [21] in our sample set; miR-10b was up-regulated more than 100-fold in two out of four AA and two out of four GBM tumors; miR-21 was up-regulated 5- to 30-fold in two out of four AA and all four GBM tumors. Also consistent with previous studies on other tumor types [11], we observed a global decrease of expression in AA and GBM tumors relative to non-neoplastic brain tissue.

**Figure 1:**
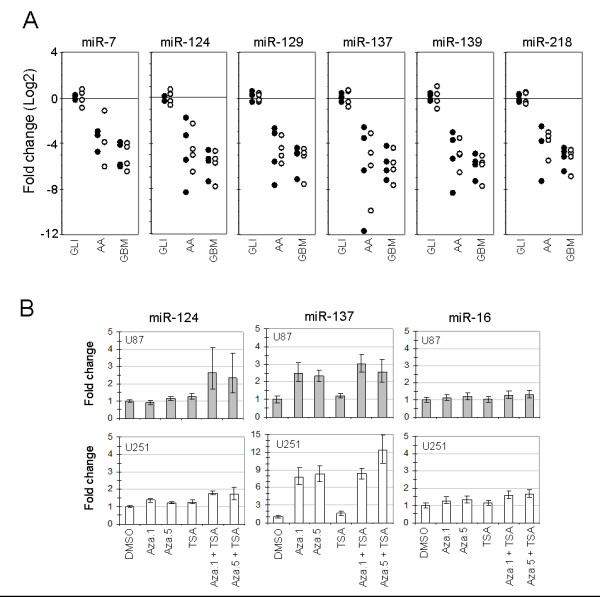
**miR-124 and miR-137 are down-regulated in anaplastic astrocytomas and glioblastoma multiformes and are up-regulated in glioblastoma multiforme cell lines following treatment with DNA demethylating agents**. (A) Expression of high-grade astrocytomas-microRNAs in individual tumor samples measured relative to let-7a (black dots) and miR-16 (white dots). Sample classes are glioses, anaplastic astrocytomas and glioblastoma multiformes. (B) Glioblastoma multiforme cell lines (U87 and U251) were treated with 5-aza-dC at 1 μM (Aza.1) or 5 μM (Aza.5) alone, trichostatin A (100 ng/ml) alone, or combinations of both agents. MicroRNA expression was measured relative to let-7a and normalized to vehicle control (dimethyl sulfoxide). Error bars represent standard deviation of triplicate polymerase chain reactions from a single experimental set. Similar results were obtained in independent experiments (see Additional file 8).

We next performed statistical analyses of our miRNA expression data to identify novel miRNAs of interest in HGAs (GBM and AA). For a summary of these analyses see Additional file 6. As shown in Table 1, we found 35 miRNAs that were significantly deregulated (P < 0.05) in AA or GBM tumors. Thirteen (37%) of these miRNAs were differentially expressed in both tumor classes relative to glioses, 16 (45%) were differentially expressed in GBM tumors only, and 6 (17%) were differentially expression in AA tumors only. We identified six miRNAs of particular interest, miR-7, miR-124, miR-129, miR-137, miR-139 and miR-218, which were down-regulated in both AAs and GBMs (Figure 1A, Additional file 8 and Table 1) at a more stringent level of significance (P ≤ 0.01). We hereafter refer to these six miRNAs as HGA-miRNAs.

**Table 1:** Differentially expressed microRNAs in anaplastic astrocytoma and/or glioblastoma multiforme tumors relative to non-neoplastic brain tissue

Glioblastoma multiforme and anaplastic astrocytoma				Glioblastoma multiforme only			Anaplastic astrocytoma only		
	Fold change glioblastoma multiforme	Fold change anaplastic astrocytoma	Ref		Fold change glioblastoma multiforme	Ref		Fold change anaplastic astrocytoma	Ref

Down-regulated				Down-regulated			Down-regulated		
**miR-7**	34	13		miR-101	4		miR-125b	153	[22]
miR-31	17	9		miR-128a	27	[22]	miR-126	7	
miR-107	14	10		miR-132	7		miR-127	149	
**miR-124**	57	26		**miR-133a**	17		miR-134	9	
miR-124b	45	27		**miR-133b**	14				
**miR-129**	43	27		miR-149	9		Up-regulated		
**miR-137**	63	66		**miR-153**	12		miR-296	11	
miR-138	27	12	[21]	miR-154*	7		miR-320	5	
**miR-139**	66	34		miR-185	5				
miR-187	6	5		miR-29b	6				
miR-203	13	4		miR-323	17				
**miR-218**	40	16		miR-328	8				
				**miR-330**	18				
Up-regulated									
miR-10b	35	75	[22]	Up-regulated					
				miR-21	7	[21,22]			
				miR-155	8				
				miR-210	7				

P-values for all miRNAs are P < 0.05 for comparisons of miRNA expression in tumors versus glioses; for miRNAs in bold P < 0.01. References indicate high-grade astrocytoma miRNA expression studies in which miRNA has been described previously.

**Figure 2:**
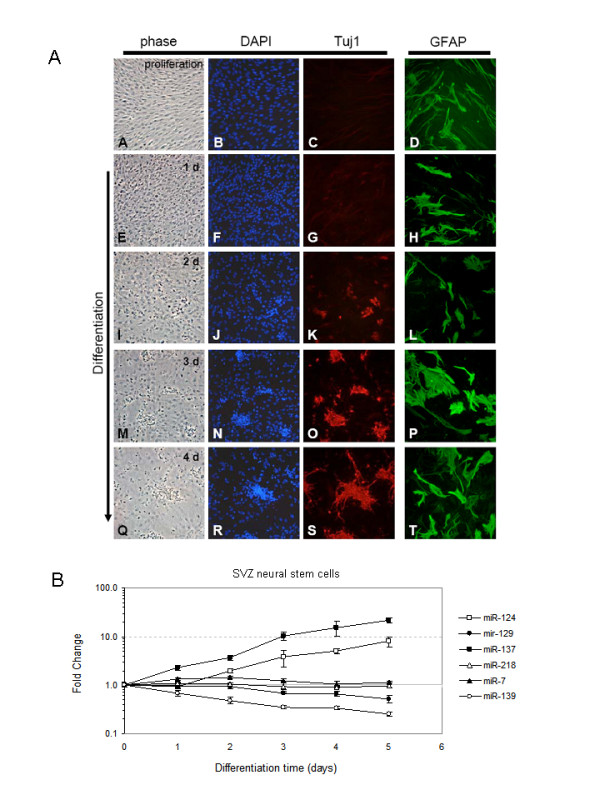
**MiRNA expression during differentiation of subventricular zone-neural stem cells**. (A) Marker expression during adult neural stem cell neurogenesis. Photomicrographs are shown of proliferating subventricular zone-neural stem cells cultures in proliferation conditions (A)-(D), and following 1 day (E)-(H), 2 days (I)-(L), 3 days (M)-(P) and 4 days (Q)-(T) of mitogen deprivation. Phase images (A), (E), (I), (M), (Q) are shown with corresponding epifluorescent images showing 4'-6-diamidino-2-phenylindole-stained nuclei (B), (F), (J), (N), (R) and Tuj1 expression (C), (G), (K), (O), (S). Glial fibrillary acidic protein expression (D), (H), (L), (P), (T) is shown in parallel cultures. (B) Expression analysis of high-grade astrocytoma-microRNAs during a 5-day differentiation time-course of subventricular zone-neural stem cells.

We observed that the majority of the HGA-miRNAs show expression changes during, or have been implicated in, differentiation of various cell lineages: miR-7 during photoreceptor differentiation [23]; miR-124 and miR-137 during erythropoiesis [24]; miR-124 and miR-218 during neuronal differentiation of embryonal carcinoma cell differentiation [25]; miR-124 during neuronal differentiation of ES cells [12]. To test whether expression of HGA-miRNAs was altered during differentiation of adult NSCs, putative precursor cells of high-grade gliomas [17], we established early passage (passage 6) cultures of SVZ-NCS as described [19]. This is a monolayer NSC culture system in which growth factor withdrawal rapidly (within 2 to 4 days) induces a large number of neuroblasts that constitute approximately 50% of the total cells after 3 to 4 days of differentiation. Consistent with previous studies [19], we observed a steady increase in the number of Tuj1+ neuroblasts over a 5-day differentiation time course (Figure 2A). In parallel cultures, we measured miRNA expression every 24 hours for 5 days (Figure 2B). Expression of miR-124 and miR-137, respectively, increased up to 8- and 24-fold, expression of miR-129 and miR-139, respectively, decreased up to 2- and 4-fold, and expression of miR-7 and miR-218 did not change appreciably.

Our differentiation studies in mNSCs suggested that growth factor signaling, which is recurrently activated in HGAs, suppresses expression of miR-124 and miR-137. It has also been shown that miR-124 expression is epigenetically suppressed in a number of tumor types including colorectal and breast cancers [26]. Further, miR-137 is closely associated with a large CpG island [27], suggesting that it may also be epigenetically silenced in tumors. We tested, therefore, whether expression of miR-124 and miR-137 could be activated in GBM cell lines following treatment with 5-aza-2'-deoxycytidine (5-aza-dC), a DNA methylation inhibitor and/or TSA, a histone deacetylase inhibitor. MiRNA-124 expression increased around 2-fold in U251 and U87 cells following combined treatment with 5-aza-dC (5 μM) and TSA (Figure 1B and Additional file 8). MiRNA-137 expression increased up to 8-fold in GBM cell lines treated with 5-aza-dC, and up to 12-fold in cells treated with both 5-aza-dC and TSA (Figure 1B and Additional file 8). Expression of both miRNAs remained relatively unchanged in cells treated with TSA alone (Figure 1B and Additional file 8). These data suggest that epigenetic modification of regulatory sequences in CpG islands may contribute to miR-124 and miR-137 silencing in GBMs.

### miR-124 and miR-137 promote neuronal differentiation of adult NSCs

**Figure 3:**
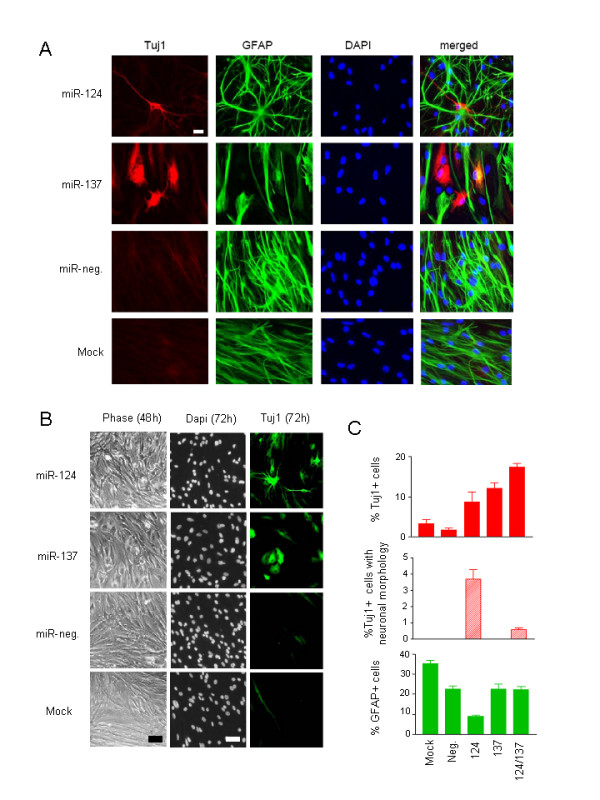
**miR-124 and miR-137 promote neuronal differentiation of subventricular zone-neural stem cells**. (A) Epifluorescent images of subventricular zone-neural stem cells 72 hours after transfection with miR-124, miR-137 and control oligonucleotide. Cells were immunostained with Tuj1 and glial fibrillary acidic protein antibodies, nuclei were counterstained with 4'-6-diamidino-2-phenylindole- and images are merged. Scale bar is 10 μm. (B) Phase contrast images of subventricular zone-neural stem cells 48 hours post-transfection by miR124 and miR137 and Tuj1 immunostaining of the same cultures 72 hours post-transfection. (C) Quantification of percentage of Tuj1+ cells, Tuj1+ cells with neuronal morphology and glial fibrillary acidic protein+ cells 72 hours after transfection with miR-124, miR-137, both miR-124 and miR-137, control oligonucleotide or transfection reagent.

To test whether up-regulation of miR-124 and miR-137 promote differentiation of adult mNSCs, we transfected proliferating mNSCs with double-stranded RNA oligonucleotides corresponding to the mature sequences of each miRNA. In each experiment, at least 80% to 90% transfection efficiencies were achieved. NSCs were maintained in proliferation medium during transfection in which cells generally have a spindle, non-neuronal morphology, with high expression of GFAP, a stem cell and astrocyte marker, but low expression of the neuronal marker Tuj1 (Figure 2A). Growth factors were withdrawn 12 to 24 hours following transfection and cells were allowed to differentiate for 72 hours. Transfection of either miR-124 or miR-137 resulted in a 5-fold increase in the numbers of cells stained with the neuronal marker Tuj1 relative to controls (Figure 3A, B and 3C). Distinct morphological changes were also apparent for each miRNA; miR-124 induced neuritic branching of the cells whereas miR-137 induced a rounded or trapezoidal cellular appearance with no neuritic outgrowth (Figure 3A and 3B). Co-transfection of miR-124 and miR-137 resulted in a near 2-fold increase in Tuj1+ cells relative to miR-124 or miR-137 transfections alone but did not promote neuronal morphologic characteristics (Figure 3C). Finally, transfection of miR-124, but not miR-137, resulted in a 2-fold decrease in the numbers of GFAP-positive cells (Figure 3A and 3C). Thus, overexpression of miR-124 and miR-137 enhances neuronal-like differentiation of adult NSCs *in vitro*.

### miR-124 and miR-137 promote neuronal differentiation of brain TSCs

**Figure 4:**
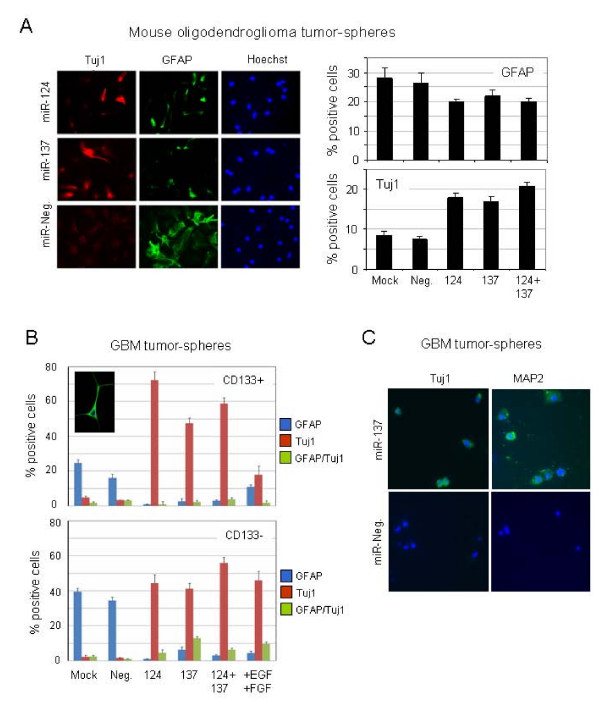
**Induction of neuronal differentiation of tumor-derived neural stem cells by miR-124 and miR-137**. (A) Epifluorescent images of tumor-derived neural stem cells 72 hours after transfection with miR-124, miR-137, control oligonucleotides and lipofectamine reagent alone. Cells were immunostained with Tuj1 and glial fibrillary acidic protein antibodies and DNA was stained with Hoechst 33258 reagent. The percentage of Tuj1- and glial fibrillary acidic protein-positive cells was quantified in each sample after transfection and staining and plotted against the total number of counted cells (*n *= 450). (C) Quantification of Tuj1+ and glial fibrillary acidic protein+ cells in primary glioblastoma multiforme cultures 10 days after transfection of miR-124, miR-137 or control oligonucleotides. The inset shows a Tuj1+ cell with neuronal morphology from a miR-124 and/or miR-137 cotransfection. (D) Immunostaining with neuronal markers Tuj1 and microtubule-associated protein 2 10 days after miR-137 or negative control miR-transfections in glioblastoma multiforme lines maintained as neurospheres.

As we observed that expression of miR-124 and miR-137 is reduced in HGAs and that miR-124 and miR-137 promote differentiation of non-neoplastic adult mNSCs, we tested next whether up-regulation of miR-124 and miR-137 could promote differentiation of brain tumor-derived stem cells. We first assessed differentiation of mOSCs derived from S100β-v-*erbB *transgenic mouse oligodendrogliomas [20]. MiRNA-124 is down-regulated in human oligodendrogliomas [28], and both miR-124 and miR-137 are down-regulated over 10-fold in S100β-v-*erbB *tumor stem cells relative to mNSCs (Additional file 7). Consistent with our observations in mNSCs, we observed a significant increase in the numbers of cells that express the neuronal marker Tuj1 following transfection with miR-124, miR-137 or a combination of both miRNAs (Figure 4A). Transfection with either miR-124 or miR-137 resulted in rounded or trapezoidal cellular morphology of Tuj1-positive cells with reduced neuritic outgrowth. We also observed that transfection of miR-124 and miR-137 reduced the numbers of GFAP-positive mOSCs (Figure 4A).

We tested next whether miR-124 and miR-137 could promote differentiation of human GBM stem cells. GBM cells were isolated from a primary tumor (SF6969) and expanded as tumor spheres in nonadherent plates. Cells were sorted using magnetic beads conjugated to an antibody against CD133, a putative marker of GBM stem cells [14,15]. Both CD133+ and CD133- cells were transfected with miR-124 and/or miR-137 and then cultured for 10 days in NBE media without growth factors. Transfection of miR-124 and/or miR-137 dramatically increased the percentage of Tuj1-positive cells, and reduced the percentage of GFAP-positive cells and in both CD133+ and CD133- GBM cell fractions (Figure 4B). Tuj1 was expressed in cells with a neuronal morphology but also in rounded cells and cells undergoing mitosis. The expression of GFAP-positive cells was confined to cells displaying typical morphology of type I and type II astrocytes.

To further investigate the role of miR-137 in neuronal differentiation of GBM cells, we assessed expression of an additional neuronal marker, MAP2, following overexpression of miR-137. Unsorted SF6969 GBM cells were transfected with miR-137 and cultured for 10 days in NBE media without growth factors. In addition to the expected increase of cells positive for Tuj-1 after 10 days, we also observed an evident increase in MAP2-postive cells following transfection of miR-137 (Figure 4C). Again, as in mNSCs and oligodendroglioma tumor spheres, miR-137 induced rounded morphology with little evidence of neuritic outgrowth (Figure 4C). Collectively, our results show that in the absence of growth factor signaling, miR-124 and miR-137 enhance neuron-like differentiation of oligodendroglial and GBM TSCs.

### miR-124 and miR-137 inhibit proliferation of GBM cell lines

**Figure 5:**
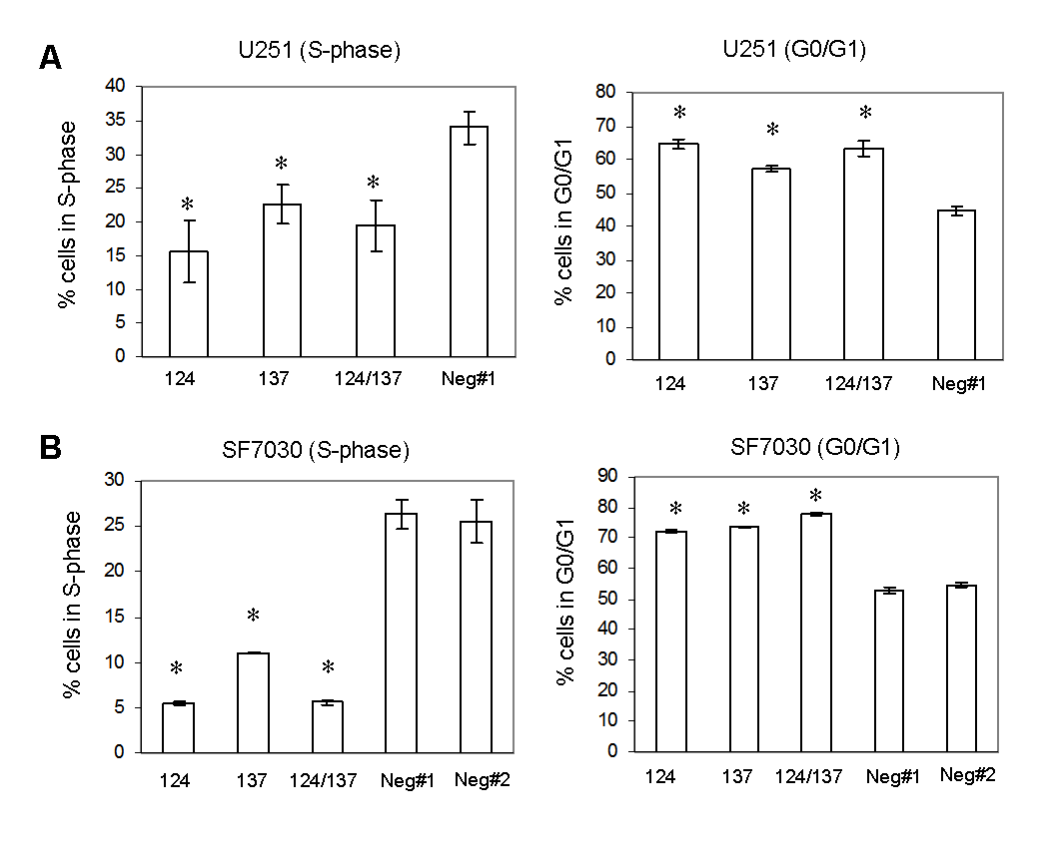
**miR-124 and miR-137 inhibit proliferation of glioblastoma multiforme stem cells and induce cell G0/G1 cycle arrest**. Cell cycle analysis was conducted by fluorescence-activated cell sorter at 48 hours after transfection of 100 nM (final total microRNA concentration) miR-124, miR-137, miR-124 and miR-137 together or negative control oligonucleotides (neg#1, neg#2) to U251 (A) and SF6969 (B) glioblastoma multiforme cells. Cells were treated with bromodeoxyuridine for 30 minutes, fixed, treated with fluorescein isothiocyanate-labeled antibromodeoxyuridine antibody and the DNA stain 7-amino-actinomycin D and subject to flow cytometry. Values represent mean ± standard deviation of replicate experiments; *P < 0.05.

Since exit from the cell cycle is required for induction of differentiation, we tested whether miR-124 and miR-137 inhibit proliferation of GBM cells. Relative to control oligonucleotides, transfection of miR-124 or miR-137 resulted in a marked reduction in the number of cells in the S-phase of the cell cycle and a marked increase in the number of cells in G0/G1 in U251 GBM cells (Figure 5A) and early passage GBM cells derived from a newly diagnosed human GBM (Figure 5B). No reproducible differences were observed for cells in G2/M of the cell cycle, or in cells undergoing apoptosis (sub G1) in any of the cell lines examined (data not shown). Our data suggest that miR-124 and miR-137 induce G0/G1 cell cycle arrest in GBM cells.

### miR-124 and miR-137 inhibit CDK6 expression and phosphorylated retinoblastoma levels in GBM cells

**Figure 6:**
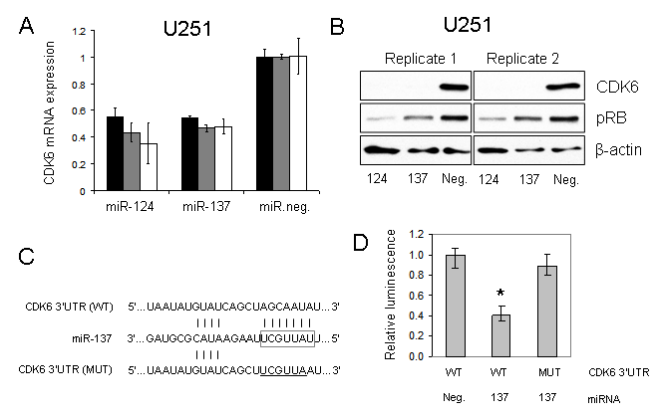
**CDK6 expression is inhibited by miR-124 and miR-137 in glioblastoma multiforme cells**. (A) Transfection of 100 nM miR-124 or miR-137 reduces cyclin-dependent kinase 6 mRNA transcript levels by 50% in U251 cells at 48 hours relative to cells transfected with 100 nM control oligonucleotide. Cyclin-dependent kinase 6 expression was determined by TaqMan relative to control genes *Gus *(black bars), *GAPDH *(gray bars) and *18S *(white bars). Values represent average +/- standard deviation of independent experiments. (B) Cyclin-dependent kinase 6 protein expression is dramatically reduced as determined by western blotting following transfection of miR-124 or miR-137. Levels of phosphorylated RB (pSer 807/811) are also markedly reduced in response to miR-124 or miR-137 transfection. (C) miR-137 sequence in relation to the pMIR-REPORT vector containing the predicted cyclin-dependent kinase 6 miR-137 binding site (wild type). Mutated bases (underlined) were also introduced into the miR-137 seed region (boxed) of the cyclin-dependent kinase 6-3'UTR (mutated). Vertical lines denote Watson-Crick base pairing. (D) Relative luminescence of U251 cells following transfection miR-137 or negative control microRNA in conjunction with wild type or mutated cyclin-dependent kinase 6 reporter constructs. *P < 0.0001.

To ascertain the molecular mechanisms by which miR-124 and miR-137 induce G0/G1 cell cycle arrest in GBM cells, we assessed expression of CDK6, a regulator of the cell cycle and differentiation (reviewed in [29]), following transfection of these miRNAs to U251 cells. CDK6 is an established target of miR-124 in HCT-116 colon cancer cells [26], a predicted target of miR-137 (TargetScan and PicTar), and has been functionally implicated in the development of multiple malignancies. In independent experiments we observed marked reductions of CDK6 transcript (Figure 6A) and CDK6 protein (Figure 6B) in response to miR-124 and miR-137 transfection. Levels of phosphorylated retinoblastoma (RB) (pSer 807/811), a known target of CDK6 [30], were also reduced in response to miR-124 and miR-137 transfection (Figure 6B).

To validate that the 3' UTR of CDK6 is a direct target of miR-137, we used a luciferase reporter system in which the predicted miR-137 binding site of CDK6 was cloned downstream of luciferase. A control reporter vector was also developed in which the seed region of the miR-137 binding site was mutated (Figure 6C). Co-transfection of U251 cells with a WT CDK6-3'UTR (CDK6-WT) reporter and the miR-137 mimic resulted in a significant decrease in luminescence (P < 0.0001) relative to cells co-transfected with CDK6-WT and a negative control miRNA mimic (Figure 6D). Mutation of the CDK6 miR-137 seed region rendered the reporter construct insensitive to inhibition by miR-137 (Figure 6D). Therefore, miR-137, in addition to miR-124, is a direct inhibitor of CDK6.

## Discussion

### Identification of miRNAs in high-grade gliomas

To identify miRNAs that are recurrently deregulated in high-grade gliomas, we used quantitative RT-PCR to profile expression of 192 miRNAs in human non-neoplastic brain tissues, AAs and GBMs. From our analyses, we identified a number of miRNAs that have been described previously in GBM tumors such as miR-10b (see [22]) and the apoptosis regulator miR-21 (see [21,22,31]). We also identified a number of miRNAs, including miR-124 and miR-137, which have not been described in prior GBM profiling studies. It remains unclear why miR-124 and miR-137 were not detected previously in GBM tumors, particularly in light of our results that show dramatic expression decreases of miR-124 and miR-137 in GBMs (and AAs) relative to non-neoplastic brain tissue, and results that show clear down-regulation of miR-124 expression in human oligodendrogliomas [28], human astroblastomas [32] and GBM cell lines [32,33].

Another notable discrepancy is that of miR-221 expression, which was not overexpressed in any of the tumors tested in our study, but was shown to be overexpressed in five out of nine of GBMs studied by Ciafre et al. [22], and has been shown to inhibit expression of the cell-cycle inhibitor p27(Kip1) in GBM cells [34]. The most obvious differences between our study and previous GBM profiling studies are: (1) the control tissues, adult glioses from epileptic surgeries, a routinely used control tissue for GBM molecular profiling studies, versus normal human fetal brain and macroscopically characterized surgical specimens from the tumor periphery [21,22]; (2) profiling technologies, TaqMan versus micro-array [21,22]. Future profiling studies on larger numbers of patient samples will help to resolve these apparent discrepancies.

Our expression analyses revealed a total of 35 miRNAs that were differentially expressed between high-grade gliomas and non-neoplastic brain tissue (P < 0.05, Table 1). A vast majority of these miRNAs were down-regulated (29 out of 35; 83%), which is consistent with observations that miRNA expression is globally down-regulated in multiple tumor types [11]. Of the 35 miRNAs, we identified six HGA-miRNAs, which were down-regulated in both AA and GBM tumors at a more stringent degree of significance (P < 0.01): miR-7, miR-124, miR-129, miR-137, miR-139 and miR-218. Although we restricted further analyses of these six miRNAs to miR-124 and miR-137 because of their elevated expression during adult NSC differentiation (Figure 1B), assessments of the other HGA-miRNAs may lead to novel insights into the biology of high-grade gliomas. Similarly, assessments of the miRNAs that were differentially expressed in AA tumors only or GBM tumors only (Table 1) may shed light on the biological differences underlying these different tumor grades.

It is important to note that our miRNA expression profiling studies were conducted at the tissue level, not at the cellular level, which has important implications for the interpretation of our results. In particular, prior work [28] has shown that miR-124 is only expressed in the neurons of adult human brains, which indicates that our observed decrease in miR-124 expression in HGAs is a likely consequence of there being relatively fewer neurons in tumor tissue compared with non-neoplastic glioses controls. While this does not change our conclusions that miR-124 and miR-137 can induce mNSC-, mOSC- and human GBM-derived stem cell (hGSC)-differentiation, it indicates that *in situ *expression analyses of miRNAs in HGAs, non-neoplastic adult brain tissue, and during fetal- and post-natal development of the mammalian central nervous system will be an important component of studies aimed at investigating the functions of miRNAs during normal brain development and tumorigenesis.

We also note that in this study we analyzed 192 of the 533 known human miRNAs that are currently described in miRBase, release 10.0 (see [35]), which reflects the rapid pace of miRNA discovery since the inception of our miRNA expression studies. It is likely that miRNAs of potential significance to brain tumor biology have not been assessed here. Examples of such miRNAs include those that show enriched expression in brain tissue such as miR-451 and miR-488 (see [32]) and miRNAs that have been implicated in the etiology of other tumor types, such as miR-346 in follicular thyroid carcinoma [36]. Therefore, comprehensive miRNA expression studies are warranted in large HGA tumor sets that are linked to clinical data, such as survival and therapeutic response in order to generate an in-depth assessment of the role of miRNAs in brain cancer etiology and therapy.

### Regulation of miR-124 and miR-137 expression

Our results reveal two potential mechanisms by which miR-124 and miR-137 may be suppressed in stem cells and/or tumor cells. The first mechanism is growth factor signaling: removal of EGF, and FGF from the culture media resulted in robust increases in miR-124 and miR-137 expression in adult NSCs. Given that activation of EGF [37], PDGF [38] and FGF [39] signaling pathways have each been implicated in gliomagenesis, it is reasonable to speculate that one mechanism by which growth factor signaling promotes brain tumor formation is through suppression of miR-124 and/or miR-137 expression and NSC/TSC differentiation. Further analyses are required to determine the relative contributions of EGF-, FGF- and PDGF-induced signaling on suppression of miR-124 and miR-137 transcription in adult NSCs and GBM tumor stem cells.

The second mechanism by which miR-124 and miR-137 expression may be suppressed in GBM stem cells is via epigenetic modification of their transcriptional regulatory sequences. Indeed, epigenetic modification of specific miRNAs in other tumor types has been reported recently. For example, miR-127, which is down-regulated in prostate, colon and bladder tumors relative to matched normal tissues, is up-regulated in cell lines derived from these tumor types following inhibition of DNA demethylation and histone deacetylase [40]. Of particular interest to our studies, miR-124 is hyper-methylated in over one-third of colon, breast, lung, lymphoma and leukemia primary tumors, and is up-regulated in breast (MCF-7) and colon (HCT-116) cancer cell lines following DNA demethylation [26]. We observed that miR-137 expression increased in GBM cell lines U87 and U251 following treatment with the DNA demethylating agent 5-aza-dC (Figure 1B). Interestingly, we did not observe an increase in miR-124 expression in either cell line following 5-aza-dC treatment. Further analyses of miR-137 and miR-124 promoter sequence methylation in primary tumors, TSCs and NSCs are warranted to establish the degree to which epigenetic mechanisms contribute to suppression of these miRNAs in HGAs.

### Regulation of differentiation and the cell cycle by miR-124 and miR-137

Previous studies have demonstrated that miR-124 is up-regulated during development of the rodent nervous system [41,42], and during neuronal differentiation of mouse ES cells [12], and mouse and human embryonal carcinoma cells [25]. Further, neuronal differentiation is enhanced following ectopic overexpression of miR-124 in mouse ES cells [12], mouse neuroblastoma cells [13], and mouse embryonal carcinoma cells [13]. Our results indicate that overexpression of either miR-124 or miR-137 promotes neuron-like differentiation of non-neoplastic adult (mNSCs), mOSCs and CD133+ hGSCs. Thus, our study is the first to implicate miR-124 in neuronal differentiation of post-natal NCSs and brain TSCs.

The ability of miR-124 to induce robust stem cell differentiation appears to be dependent on cell type, developmental timing and other, as yet unidentified, factors. For example, in mouse neuroblastoma cell lines CAD and Neuro2a, ectopic up-regulation of miR-124 alone is sufficient to induce neuron-like differentiation, whereas in mouse embryonic carcinoma cells (P19), miR-124 enhances neuronal differentiation only in the presence of retinoic acid, an established inducer of P19 neuronal differentiation [13]. Investigations of miR-124 expression and function during development of the embryonic chick spinal cord have determined that the proneural activity of miR-124 is, at best, subtle [43,44], suggesting that additional factors- and/or signals are required for robust neurogenesis at this developmental stage. Our studies show that miR-124 and miR-137 enhance neurogenesis of mNSCs, mOSCs and hGSCs in the absence of growth factor signaling. Although we have not tested whether miR-124 and miR-137 alone can induce differentiation of the various stem cells tested in this study, transfection of miR-124 or miR-137 alone was sufficient to induce G1 cell cycle arrest in standard GBM cell lines (Figure 5A). However, cell cycle arrest was more pronounced in miR-124- and miR-137-transfected GBM cells (SF6969) that were deprived of growth factors (Figure 5B). Overall, the most robust effects of miR-124 and miR-137 overexpression on cellular differentiation and proliferation were observed in growth factor-deprived human cells (Figures 4B and 5B). Collectively, our results suggest that while miR-124 and miR-137 have the capacity to induce alone cell cycle arrest and differentiation in human GBM cells and stem cells, abrogation of growth factor signaling enhances their capacity to do so. Additional studies will be required to address this hypothesis, and incorporation of additional GBM and oligodendroglioma-neurosphere lines will be required to address the general applicability of our results in relation to the biology and therapeutics of these diseases.

Recent studies have begun to shed light on the molecular mechanisms by which miR-124 regulates differentiation and proliferation. For example, miR-124 directly targets PTBP1 (PTB/hnRNP I) mRNA, a global repressor of alternative pre-mRNA splicing in non-neuronal cells, resulting in the transition from non-neuronal- to neuronal-specific alternative splicing patterns [13]. miR-124 also directly targets and suppresses expression of small C-terminal domain phosphatase 1 (SCP1), an inhibitor of neuronal gene expression [44]. Finally, miR-124 overexpression in HCT-116 colon cancer cells inhibits the expression of CDK6, an established target of miR-124 (see [26]). Our studies revealed that miR-137, as well as miR-124, inhibited expression of CDK6, a predicted target of both miRNAs. Further, as with miR-124a (see [26]), our results show that miR-137 is a direct inhibitor of CDK6. Overexpression of miR-124 or miR-137 also reduced the expression of phosphorylated RB (Figure 6B), a downstream target of CDK6 [30]. It is interesting to note that CDK6 is known to regulate both cell cycle progression and differentiation (reviewed in [29]), suggesting that mir-124- and miR-137-mediated inhibition of CDK6 may, in part, account for the observed effects on GBM cell proliferation and differentiation in this study. Further investigations are needed to define the relationship between CDK6 down-regulation and cell cycle arrest and/or differentiation in GBM stem cells, and to identify and characterize additional miR-124 and miR-137 target genes.

### Therapeutic potential of miR-124 and miR-137

The ability of miR-124 and miR-137 to induce potent antiproliferative and prodifferentiation effects in CD133+ and CD133- human GBM cells suggests their potential value for treatment of this disease. RNAi-based therapeutics holds great promise for the development of entirely novel therapeutic strategies for disease treatment [45], and early phase clinical trials using siRNAs are currently underway [46]. While delivery of siRNAs or miRNAs to the central nervous system is particularly challenging because of the blood brain barrier, a number of promising strategies have been developed recently to circumvent this problem. These include intranasal delivery of oligonucleotides [47], lipid encapsulation and targeted delivery of nucleic acids [48,49], and direct administration of therapeutic agents to brain tumor tissues by convection-enhanced delivery [50,51]. Further testing of miR-124 and miR-137 in pre-clinical models of GBM [52,53] in conjunction with various delivery strategies will help define their ultimate therapeutic potential for treatment of GBM.

## Conclusion

We have investigated the role of miRNAs in adult human HGAs and hGSCs and in adult mNSCs and mOSCs. Our studies showed, for the first time to the best of the authors' knowledge, that miR-124 and miR-137: (1) are expressed at significantly lower levels in GBM tumors relative to non-neoplastic brain tissue; (2) are up-regulated during neuronal differentiation of adult mNSCs induced by growth factor withdrawal; (3) promote neuronal-like differentiation of growth-factor-deprived mNSCs, mOSCs and hGSCs; (4) promote G0/G1 cell cycle arrest in GBM cells and growth-factor-deprived hGSCs; (5) inhibit expression of CDK6 mRNA, CDK6 protein and phosphorylated RB in GBM cells. These results suggest that targeted delivery of miR-124 and/or miR-137 to GBM tumor cells may be therapeutically valuable for GBM disease treatment.

## List of abbreviations

AA: anaplastic astrocytomas; bFGF: basic fibroblast growth factor; CD: cluster of differentiation; CDK6: cyclin-dependent kinase 6; DMEM: Dulbecco's Modified Eagle's Medium; EDTA: ethylene diamine tetraacetic acid; EGF: epidermal growth factor; ES: embryonic stem; FCS: fetal calf serum; FGF: fibroblast growth factor; GBM: glioblastoma multiforme; GFAP: glial fibrillary acidic protein; HGA: high grade astrocytoma; hGSC: human GBM-derived stem cell; MAP2: microtubule-associated protein 2; miRNA: microRNA; mNSC: mouse neural stem cells; mOSC: mouse oligodendroglioma tumor stem cells; mRNA: messenger RNA; MUT: mutated; NCS: neural stem cells; PDGF: platelet-derived growth factor; RB: retinoblastoma; RT-PCR: reverse transcriptase polymerase chain reaction; siRNA: short interfering RNA; SVZ: subventricular zone; TSA: trichostatin A; TSC: tumor stem cell; UCSF: University of California San Francisco; WHO: World Health Organization; WT: wild type.

## Competing interests

David Ginzinger, a collaborator and co-author of the manuscript, was an employee of Applied Biosystems at the time the experiments were conducted. The miRNA TaqMan assays were provided by David Ginzinger.

## Authors' contributions

JS performed the miRNA expression studies, proliferation assays in GBM cells, CDK6 and Rb Western blotting, luciferase reporter assays, developed the miRNA transfection protocols and helped to draft the manuscript. DAL designed, implemented, interpreted and described experiments involving SVZ-NSCs. CP designed, implemented, interpreted and described experiments involving mOSCs. AIP designed, implemented, interpreted and described experiments involving hGSCs. AKM designed, implemented, interpreted and described demethylation and deacetylation experiments. MY assisted with design, implementation and interpretation of miRNA expression studies. SRV provided a histopathological review of human tissues and critical revision of the manuscript. DGG provided design and support for the miRNA expression analyses. CDJ provided overall conception and design support and critical revision of the manuscript. JFC provided design and analyses support for the demethylation and deacetylation experiments and critical revision of the manuscript. GB provided design and analyses support for the differentiation studies of mOSCs and critical revision of the manuscript. WAW provided design and analyses support for the differentiation studies of hGSCs and critical revision of the manuscript. AA–B provided design and analyses support for the miRNA expression and differentiation studies of SVZ-NSCs and critical revision of the manuscript. JGH conceived the study, participated in its design and coordination and drafted the manuscript. All authors read and approved the final manuscript.

## Pre-publication history

The pre-publication history for this paper can be accessed here:



## Supplementary Material

Additional file 1 Characteristics of the patient tissues used in this study. Click here for file

Additional file 2 Validation of miR-124 expression and function in glioblastoma multiforme cells. Click here for file

Additional file 3 **Supplementary methods**. This pdf file provides further details related to the methods used in this study. Click here for file

Additional file 4 Log2 fold change of microRNAs in non-tumor brain samples (glioses), anaplastic astrocytomas and glioblastoma multiforme tumors. Click here for file

Additional file 5 Validation of let-7a and miR-16 as appropriate control miRNAs in primary tumor samples and neural stem cells. Click here for file

Additional file 6 Statistical comparisons of miRNA expression in glioblastoma multiforme and anaplastic astrocytoma tumors versus non-tumor brain tissue (gliosis). Click here for file

Additional file 7 Assessment of miR-124a and miR-137 expression in mouse oligodendroglial stem cells. Click here for file

Additional file 8 miR expression in glioblastoma multiforme cell lines following treatment with DNA demethylating and deacetylating agents Click here for file
