# Supplementary material for: miR-124 and miR-137 inhibit proliferation of glioblastoma multiforme cells and induce differentiation of brain tumor stem cells
Source: BMC Med. 2008 Jun 24;6:14. doi: 10.1186/1741-7015-6-14 (PMC2443372; doi:10.1186/1741-7015-6-14)
Supplement: Additional file 2 — Validation of miR-124 expression and function in glioblastoma multiforme cells. [file 1741-7015-6-14-S2.pdf]

A

B

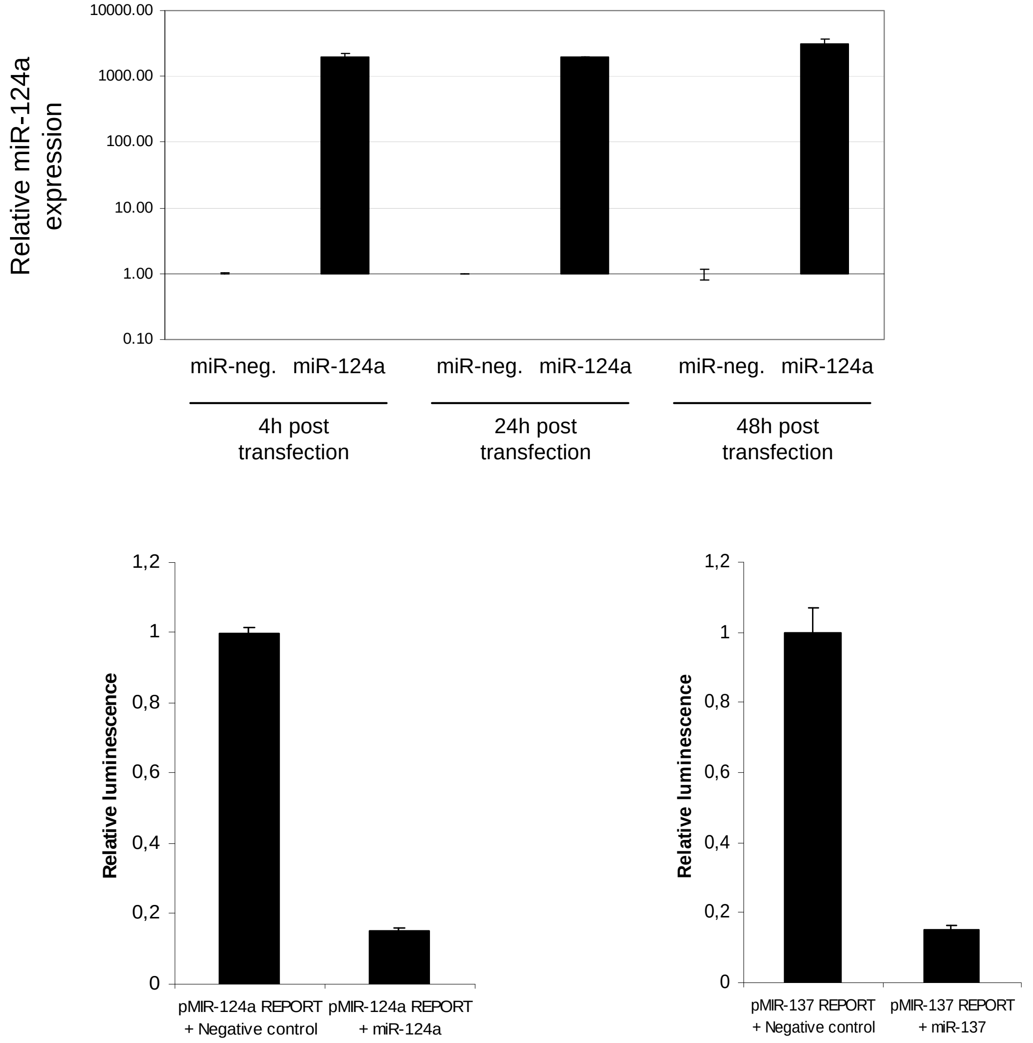

C

Oligonucleotide	Sequence
miR-137 fw	5’- AGCTTAACTGTTATTGCTTAAGAATACGCGTAGGCGTCTATGCCGGCCTATGAGCTCCCTCA
miR-137 rv	5’- CTAGTGAGGGAGCTCATAGGCCGGCATAGACGCCTACGCGTATTCTTAAGCAATAACAGTTA
miR-124a fw	5’- AGCTTAACTGTTTAAGGCACGCGGTGAATGCCAGCGTCTATGCCGGCCTATGAGCTCCCTCA
miR-124a rv	5’- CTAGTGAGGGAGCTCATAGGCCGGCATAGACGCTGGCATTCACCGCGTGCCTTAAACAGTTA

Additional file 2 (Supplementary figure 1) - Validation of miR-124 expression and function in GBM cells. (A) miR-124 expression measured by TaqMan at 4h-48h following transfection of 100nM miR-124, or negative control microRNA (miR-neg), to U251 GBM cells (B) Expression of luciferase from the pMIR-REPORT vector (Ambion) containing a miR-124 binding site (pMIR-124 REPORT) is highly abrogated in the presence of miR-124 oligonucleotide mimics; Luciferase expression from pMIR-137 REPORT is highly abrogated in the presence of miR-137 oligonucleotide mimics. To generate pMIR-124a REPORT and pMIR-137 REPORT vectors, direct match miRNA target sites for miR-124a and miR-137 were generated by annealing complementary oligos (C), and then cloned into the HindIII and SpeI restriction sites of the pMIR-REPORT vector. A total of 200ng reporter vector and 50nM miR-124a, miR-137 or cel-miR-67 mimics were co-transfected into HEK 293T cells using siPORT NeoFX transfection Agent (Ambion). Luciferase expression was measured 48 hours post-transfection using the Xenogen Bioimaging luminescence system (IVIS 100).
