# Supplementary material for: miR-124 and miR-137 inhibit proliferation of glioblastoma multiforme cells and induce differentiation of brain tumor stem cells
Source: BMC Med. 2008 Jun 24;6:14. doi: 10.1186/1741-7015-6-14 (PMC2443372; doi:10.1186/1741-7015-6-14)
Supplement: Additional file 3 — Supplementary methods. This pdf file provides further details related to the methods used in this study. [file 1741-7015-6-14-S3.pdf]

SUPPLEMENTARY METHODS

Quantitative RT-PCR and data analysis

Total RNA was extracted using the miR-Vana RNA isolation system (Ambion, Austin TX). miR expression was measured by quantitative RT-PCR using TaqMan® miR assays human panel-early access kit following manufacturer's directions (Applied Biosystems, Foster City CA). Briefly, 10ng of total RNA was used in separate RT reactions primed with miR specific oligonucleotides. For each RT, we determined cycle threshold (Ct) in duplicate PCR reactions containing miR-specific forward primers and Taqman probes, and set an upper Ct limit of 35 on all measurements (i.e. we considered samples with Cts > 35 as not expressed). We calculated the average Ct of duplicate measurements for each miR, and excluded those with standard deviations >2 (<1% of miRs in all samples). We calculated fold change in expression using the comparative Ct ($\Delta\Delta$Ct) method, using let-7a and miR-16 as the endogenous control miRs and the average ΔCt of the 4 glioses as the calibrator. We used the same approach to measure miR expression in neural stem cells and GBM cell lines except that PCR reactions were run in triplicate.

Statistical Analyses

Statistical analyses were separately performed on log2 transformed fold change expression data using freely available R language. We focused on miRs that showed consistent fold changes (Spearman correlation > 0.5) across samples between the let-7a and miR-16 datasets; this represented the majority (77%) of

miRs. Because of the high variability in expression within a group, the limma package in Bioconductor was used to compare the three types of primary tissues (glioses, AA, and GBM). Moderated t-statistics and F-statistics (1) based on empirical Bayes method of shrinkage of standard errors towards a common value were obtained from linear models and p-values were adjusted by controlling the false discovery rate. Changes were considered significant if the false discovery rate was less than 0.05. To identify differentially expressed miRNAs between glioses vs. AA and glioses vs. GBM samples, a linear 1-way layout was fit with the glioses as the reference and moderated t-statistics obtained. To make all pair-wise comparisons between the three types, moderated F-statistics were extracted from 1-way ANOVA fit. Differentially expressed miRNAs between AA and GBM types were also identified using moderated t-statistics.

<u>Cell line transfections</u>

For each of the cell lines assessed in our study 80-90% transfection efficiencies were achieved. This was determined by a visual assessment of the cells under a fluorescent microscope following transfection with control miRNA mimics tagged with a Cy3 fluorophore.

<u>Establishment and growth of mouse SVZ–NSCs</u>

SVZ microdissections from 2-month-old CD-1 mice (Charles River Laboratories) were dissociated to a single cell suspension with 0.25% Trypsin, 0.5mM EDTA and gentle trituration. Cells were cleared on a 22% Percoll (Sigma) step-gradient (2) and grown in proliferation medium (DMEM/F12/N2, 5% FCS, 20 ng/ml EGF,

20 ng/ml bFGF, 35 ug/ml bovine pituitary extract (all media and supplements from Invitrogen, Inc.). Non-attached cells were collected after 1d and re-plated into a 35mm tissue culture dish (Corning). After 7-10d, the plate was hyperconfluent with SVZ-NSCs, and these were routinely passaged 1:2 with 0.25% Trypsin, 0.5mM EDTA. Cells were passaged at least 6 times before use in experiments. Media was half-changed every 2d, and completely changed every 4 days. Differentiation of SVZ-NSCs for the miR expression time course was induced by removing the EGF, FGF, and FCS from the media (3).

<u>Immunocytochemistry and cell culture quantification</u>

SVZ-NSC and S100β-v-*erbB*-TSC cultures were fixed with 4% paraformaldehyde for 20 minutes at 25°C; GBM cultures were fixed in 2% paraformaldehyde for 10 min at 4°C. After 2-3 washes in phosphate buffered saline (PBS), GBM cells were pre-blocked for 30 min in PBS with 5% donkey serum and 0.1% triton X-100. Primary antibodies (Tuj1, 1:500, Covance Inc.; GFAP, rabbit polyclonal, 1:500, Dako Inc.; MAP2ab, 1:500, Sigma) were incubated overnight at 4°C in PBS with 10% goat serum, 0.3% Triton X-100 (SVZ-NSCs, S100β-v-*erbB* TSCs) or in PBS with 5% donkey serum and 0.1% triton X-100 (GBM TSCs). Secondary antibodies (Alexa488 donkey anti-mouse and Alexa594 donkey anti-rabbit were incubated at 25°C for 1-2 h at 1:500-1:1000 dilution. Nuclei were counterstained with Hoechst 33258 (Molecular Probes) or DAPI (Sigma) and mounted with Aquamount medium. For quantification, five non-overlapping high-power (400x) fields of view were captured on a digital camera with OpenLab software (Agilent Inc.) or ImagePro software (Technical Instruments), and immunostained cells were manually counted in Photoshop (Adobe) or ImageJ (NIH).

Cell cycle analysis

Unsorted U251 and SF7030 GBM cells were plated in six-well plates and transfected with 100 nM microRNA mimics as described above. For SF7030 cells, growth factors were removed 4h post-transfection. At 48 h post-transfection, the cells were labeled with 10 μM BrdU for 40min, then stained with FITC-conjugated anti-BrdU antibodies and 7-amino-actinomycin (a general DNA stain) using the FITC BrdU Flow Kit following manufacturers recommendations (BD Pharmingen San Diego, CA). Stained cells were then quantified by flow cytometry using a FACSCalibur multicolor flow cytometer and CellQuest Pro software (BD Biosciences; San Diego, CA).

Immunoblotting

Cleared cell lysate was electrophoretically resolved on denaturing sodium doedecyl sulfate (SDS)-polyacrylamide gels (4%–12%) and transferred to polyvinylidene difluoride membranes (PVDF; Millipore). The membrane was incubated with an antibody against CDK6 (1:1000; #3136 Cell Signaling, Temecula, CA) and Phospho-Rb (1:1000; #9308 Cell Signaling Technology, Temecula, CA), using standard techniques. An antibody against ß-actin (1:5000; Sigma, St. Louis, MO) was used as a loading control. Bound antibodies on immunoblots were detected by chemiluminescent (ECL, Amersham).

1. Smyth, G. K. Linear models and empirical bayes methods for assessing differential expression in microarray experiments. Stat Appl Genet Mol Biol, *3:* Article3, 2004.
2. Lim, D. A., Tramontin, A. D., Trevejo, J. M., Herrera, D. G., Garcia-Verdugo, J. M., and Alvarez-Buylla, A. Noggin antagonizes BMP signaling to create a niche for adult neurogenesis. Neuron, *28:* 713-726, 2000.
3. Scheffler, B., Walton, N. M., Lin, D. D., Goetz, A. K., Enikolopov, G., Roper, S. N., and Steindler, D. A. Phenotypic and functional characterization of adult brain neuropoiesis. Proc Natl Acad Sci U S A, *102:* 9353-9358, 2005.
