# Supplementary material for: miR-124 and miR-137 inhibit proliferation of glioblastoma multiforme cells and induce differentiation of brain tumor stem cells
Source: BMC Med. 2008 Jun 24;6:14. doi: 10.1186/1741-7015-6-14 (PMC2443372; doi:10.1186/1741-7015-6-14)
Supplement: Additional file 5 — Validation of let-7a and miR-16 as appropriate control miRNAs in primary tumor samples and neural stem cells. [file 1741-7015-6-14-S5.pdf]

A

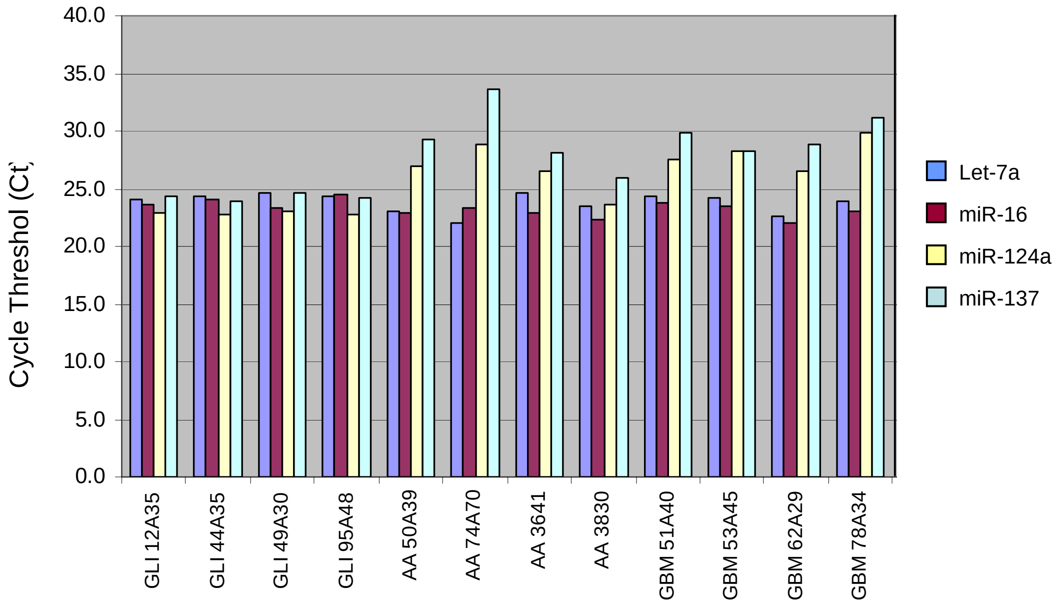

B

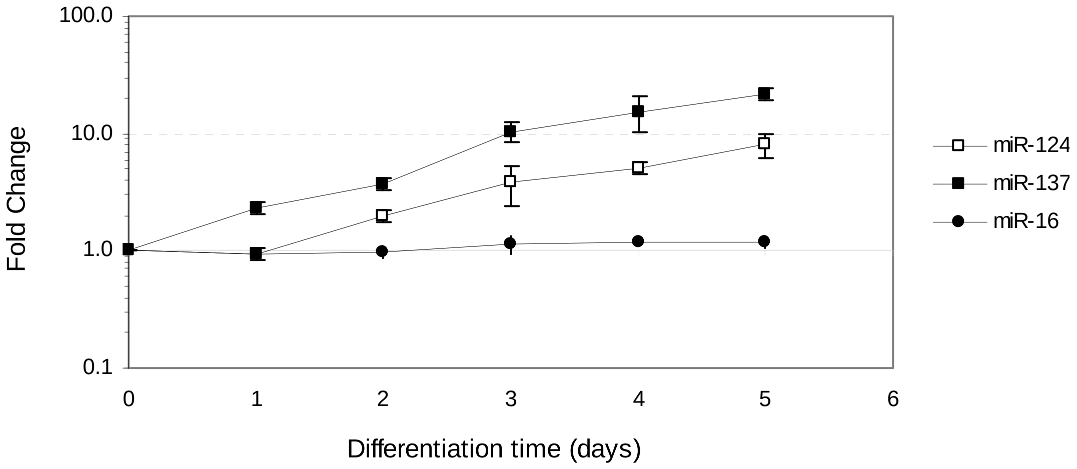

Additional file 5 (Supplementary figure 2). Validation of Let-7a and miR-16 as appropriate control miRs in primary tumor samples and NSCs (A) TaqMan cycle threshold (Ct) for miR-16, let-7a, miR-124, and miR-137 is plotted for primary tissue samples. Let-7a and miR-16 show little variation in Ct across samples relative to miR-124 and miR-137. Equal amounts of RNA (10ng; based on spectrophotometric measurements) were put into each RT reaction. (B) Expression of miR-16, relative to let-7a, does not change during differentiation of NSCs, whereas expression of miR-124a and miR-137, relative to let-7a, does.
