# Supplementary material for: miR-124 and miR-137 inhibit proliferation of glioblastoma multiforme cells and induce differentiation of brain tumor stem cells
Source: BMC Med. 2008 Jun 24;6:14. doi: 10.1186/1741-7015-6-14 (PMC2443372; doi:10.1186/1741-7015-6-14)
Supplement: Additional file 7 — Assessment of miR-124a and miR-137 expression in mouse oligodendroglial stem cells. [file 1741-7015-6-14-S7.pdf]

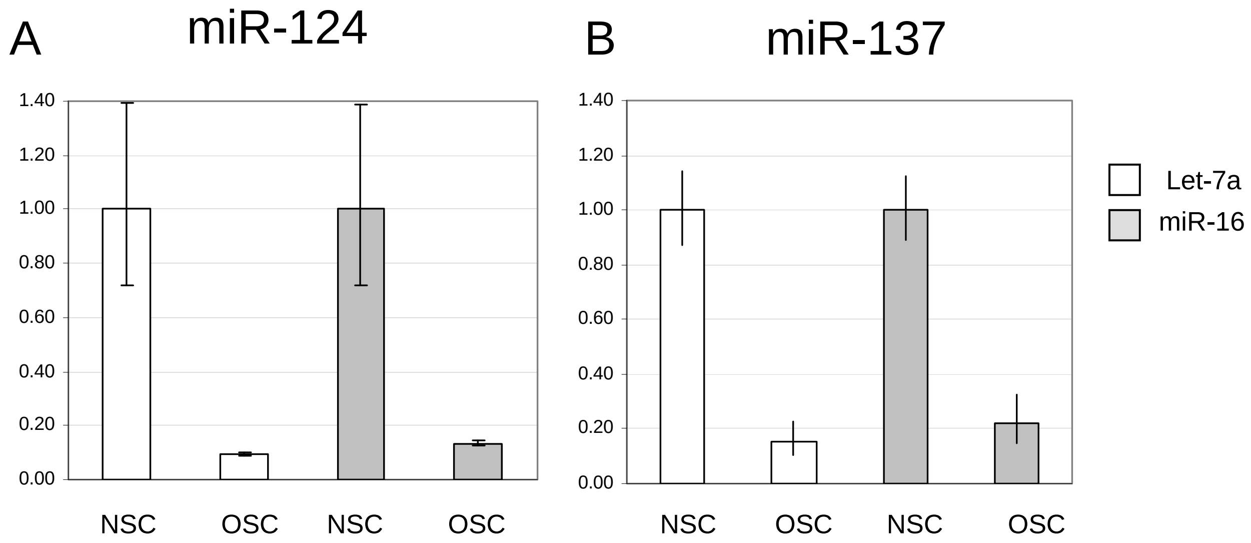


**Additional file 7 (Supplementary figure 3) Assessment of miR-124a and miR-137 expression in mouse oligodendroglial stem cells.** miR-124 (A) and miR-137 (B) are downregulated in mouse oligodendroglial stem cells (OSCs) relative to wild type mouse neural stem cells (NSCs). miR-124 and miR-137 expression was measured relative to control miRs let-7a and miR-16.
