# Supplementary material for: miR-124 and miR-137 inhibit proliferation of glioblastoma multiforme cells and induce differentiation of brain tumor stem cells
Source: BMC Med. 2008 Jun 24;6:14. doi: 10.1186/1741-7015-6-14 (PMC2443372; doi:10.1186/1741-7015-6-14)
Supplement: Additional file 8 — miR expression in glioblastoma multiforme cell lines following treatment with DNA demethylating and deacetylating agents [file 1741-7015-6-14-S8.pdf]

Experiment A

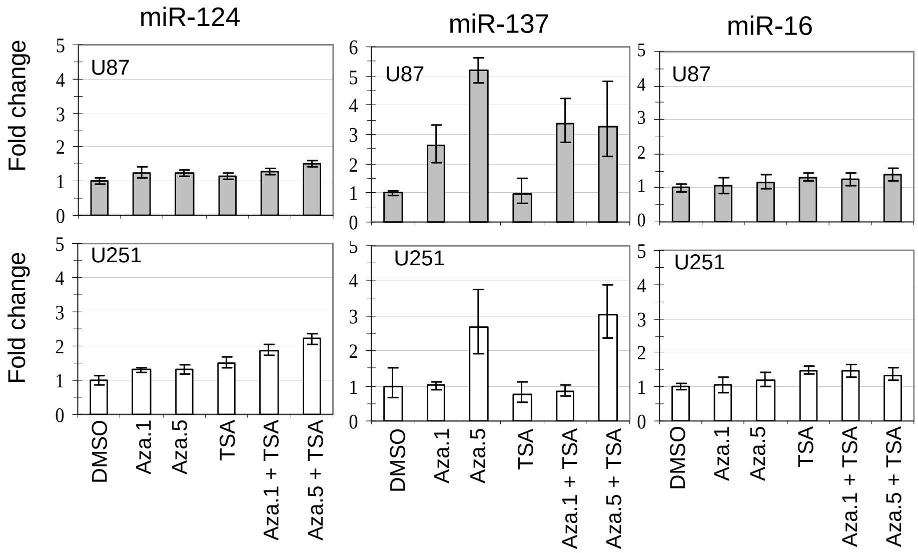

Experiment B

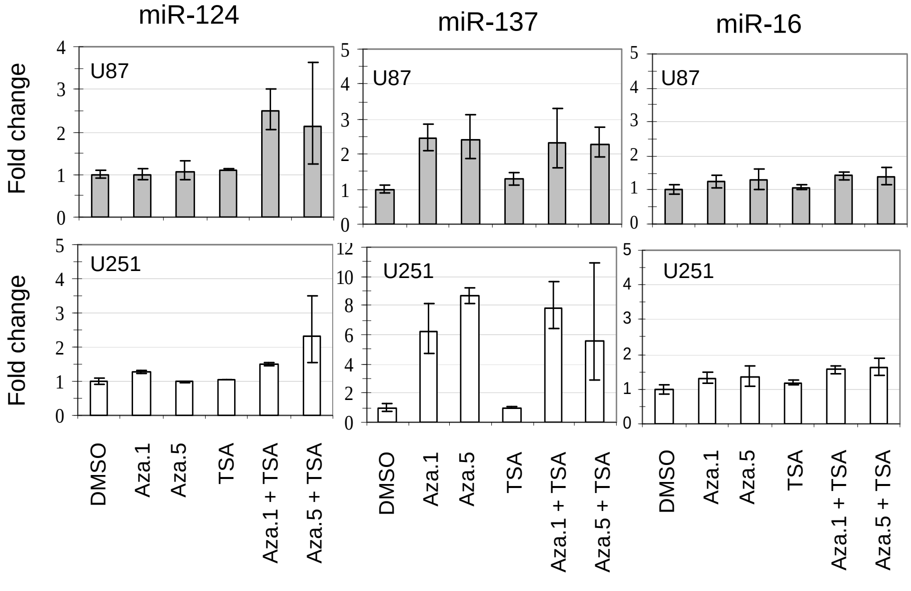

Additional file 8 (Supplementary figure 4). miR expression in GBM cell lines following treatment with DNA demethylating and deacetylating agents. miR expression in GBM cell lines (U87 and U251) treated with 5-aza-dC at 1uM (Aza.1) or 5uM (Aza.5) alone, TSA (100 ng/ml) alone, or combinations of both agents. miR expression was measured relative to let-7a , and normalized to vehicle control (DMSO). Error bars represent SD of triplicate PCR reactions from a single experimental set. Results from two independent experiments are shown.
